# The Effect of a Simulated Basketball Game on Players’ Sprint and Jump Performance, Temperature and Muscle Damage

**DOI:** 10.1515/hukin-2015-0045

**Published:** 2015-07-10

**Authors:** Vytautas Pliauga, Sigitas Kamandulis, Gintarė Dargevičiūtė, Jan Jaszczanin, Irina Klizienė, Jūratė Stanislovaitienė, Aleksas Stanislovaitis

**Affiliations:** 1 Lithuanian Sports University; Department of Education Science, Kaunas University of Technology. Kaunas.; 2 Institute of Sports Science and Innovations, Lithuanian Sports University. Kaunas.; 3 Gdansk University of Physical Education and Sport, Poland.; 4 Department of Coaching Science, Lithuanian Sports University.

**Keywords:** power, fatigue, potentiation, hyperthermia, creatine-kinase activity

## Abstract

Despite extensive data regarding the demands of playing basketball, the relative importance of factors that cause fatigue and muscle potentiation has been explored only tentatively and remains unclear. The aim of this experimental field study was to assess changes in leg muscle power and relate these changes to body temperature modifications and indices of exercise-induced muscle damage in response to a simulated basketball game. College-level male basketball players (n=10) were divided into two teams to play a simulated basketball game. Ten-meter sprint and vertical counter-movement jump tests, core body temperature and creatine-kinase activity were measured within 48 h after the game. The participants’ body temperatures increased after a warm-up (1.9%, p<0.05), continued to increase throughout the game, and reached 39.4 ± 0.4ºC after the fourth quarter (p<0.05). The increase in temperature during the warm-up was accompanied by an improvement in the 10-meter sprint time (5.5%, p<0.05) and jump height (3.8%, p<0.05). The players were able to maintain leg power up to the fourth quarter, i.e., during the major part of the basketball game. There was a significant increase in creatine-kinase at 24 h (>200%, p<0.05) and 48 h (>30%, p<0.05) after the game, indicating damage to the players’ muscles. The basketball players’ sprint and jump performance appear to be at least in part associated with body temperature changes, which might contribute to counteract fatigue during the larger part of a basketball game.

## Introduction

Playing efficiency in team sports strongly depends on athletes’ physical capacities (Cortis et al., 2011). In a basketball game, lower-body muscle power, which is determined by jump height and acceleration during short sprints (Castagna et al., 2007; Ben Abdelkrim et al., 2007; Erculj et al., 2010; Alemdaroğlu, 2012), is of great importance. These abilities are essential because a single basketball game includes up to 50 jumps per player, while approximately 10% of the movements during a game are sprints that cover 10–20 meters (Drinkwater et al., 2008). The ability to move quickly and jump as high as possible (when reaching) determines player’s performance and the quality of other technical actions that are important in basketball: fast breaks, rapid transitions from defense to offense, jump shots, fighting for the rebound and defense activities.

It is well known that fatigue is experienced when physical exercise begins during competition or training sessions. However, the effect of fatigue is masked for a while because functional capacities are determined by the coexistence of factors that cause fatigue and muscle potentiation. Fatigue depends mainly on energy depletion, metabolite accumulation and the inhibition of Ca2+ release by the sarcoplasmic reticulum (Enoka and Duchateau, 2008), whereas the potentiation mechanisms include mechanical muscle activation and temperature effects. In addition to fatigue and potentiation, basketball players experience exercise-induced muscle damage (EIMD), which is caused by movements that contain an eccentric component (e.g., when the player lands after a vertical jump or decelerates after a sprint) and leads to marked performance deterioration (Chatzinikolaou et al., 2014). The well-documented symptoms of exercise-induced muscle damage include disruption of the intracellular muscle structure, sarcolemma and extracellular matrix (Lauritzen et al., 2009), extended impairment of muscle function during contractions (Byrne et al., 2004; Skurvydas et al., 2010), protein leakage from injured muscle fibers, acute inflammation reaction, delayed-onset muscle soreness, stiffness and swelling (MacIntyre et al., 2001; Skurvydas et al., 2011).

The dominance of fatigue or potentiation factors depends on the exercise type, its intensity and duration of recovery between sets (Fowles and Green, 2003; Baudry and Duchateau, 2004). Despite extensive evidence regarding the demands of a basketball game, the relative importance of factors that cause fatigue and muscle potentiation has been explored tentatively and remains unclear. Therefore, the purpose of the present investigation was to assess the changes in lower limb muscle power in response to a simulated basketball game and relate these changes to body temperature modifications and indices of exercise-induced muscle damage. It was hypothesized that short sprint and jump changes performance coincide with temperature increases and that exercise-induced muscle damage significantly affects lower limb power indicators during post-game recovery.

## Material and Methods

### Subjects

The subjects were college-level male basketball players (n=10; age [mean ± standard deviation (SD)], 21.5 ± 1.7 years; body mass 83.5 ± 8.9 kg; body height 192.5 ± 5.4 cm). Their training experience ranged from 7 years to approximately 10 years. The experiment was performed during the pre- season period, during which the basketball players trained for 2 hours 4–5 times per week. Time was shared to approximately 80% for players’ conditioning and 20% for technical skills development at this phase of the annual training cycle. The team had ranked second in the college league and first in the Lithuanian National Basketball division in current year competitions.

The players avoided intensive exercise 48 h before the testing procedures. Each subject read and signed a written informed consent form consistent with the principles outlined in the Declaration of Helsinki. Ethical approval was granted by the Lithuanian University of Health Scienses institutional review board.

### Experimental design

After body height, mass, temperature and creatine-kinase (CK) activity were measured, the subjects completed a standardized questionnaire indicating their age and training experience. Subsequently, they participated in a 20 min aerobic-type warm-up that consisted of slow jogging (10 min), dynamic active stretching exercises (5 min), slow dribbling with shooting the ball (2 min) and free throws (3 min). The participants were then divided into two teams by the coach. The criteria for team assignment were the basketball performance level and playing position. The two teams played a simulated game that consisted of four 10 min quarters with a 15 min break at half time and 8-min breaks after the first and the third quarters. The players usually had a 2 min break for rest after the first and the third quarters, but the subjects in the present study rested for 2 min during those breaks and then performed tests for 6 min. The game involved official umpires, and took place on an indoor basketball court. Player substitutions were not allowed, and the players stayed in the game even when they had five fouls. There was only a four-point difference in the final game score between the two teams, which equaled 82:86. Ten-meter sprint and vertical counter-movement jump tests were performed before and after the warm-up, immediately after each of the four quarters and 20 min, 24 h and 48 h after the game. Core body temperature was measured before and after the warm-up, immediately after the fourth quarter, 24 h and 48 h after the game. CK activity was recorded 24 h and 48 h after the game. The participants received verbal feedback about their performance after each test and were encouraged to apply maximal effort during each test.

### Test protocols

*Ten-meter sprint.* Running time was recorded using the Powertimer Testing System (New Test, Oulu, Finland). Photo-sensing elements connected to an electronic chronometer were placed 10 m apart. The starting position was 70 cm from the first photo-sensing element. Two trials were conducted with a recovery time of approximately 2 min between them. The best result was used for further analysis. The intra-class correlation coefficient (ICC) for this test was established previously (0.95; Kamandulis et al., 2013).

*Counter-movement jump without arm swing.* The participants performed the vertical jump on a contact mat (Powertimer Testing System, New Test, Oulu, Finland) starting from an upright standing position with preliminary downward movement to a knee angle of approximately 90° with an arm swing. Three trials were performed with 20 s of rest between each trial. The best result was used for analysis. If the third trial result was the best, one additional trial was carried out. The height of the jumps was calculated by applying the following equation: H = 1.226 × Tf^2^ (m), where Tf = flight time (s; Bosco et al., 1982). The best result was used for further analysis. The ICC for this test was established previously (0.95; Kamandulis et al., 2013).

*Plasma CK activity.* Approximately 0.5 mL of mixed capillary blood was drawn from each subject’s finger. The samples were centrifuged immediately and analyzed for CK activity using the Spotchem^™^ EZ SP-4430 biochemical analyzer and the manufacturer’s soft reagent strips (Arkray Factory, Inc., Shiga, Japan). The normal reference range of plasma CK activity for humans using this method is between 56 and 244 IU·L^−1^ according to the manual that was provided with the analyzer.

*Body temperature.* Rectal body temperature was measured using a thermocouple (Rectal Probe, Ellab, Hvidovre, Denmark; accuracy ± 0.01ºC) that was inserted to a depth of 12 cm past the anal sphincter. Each subject placed the rectal thermostat sensor independently.

### Statistical analyses

Descriptive data are presented as the mean ±SD. The Kolmogorov–Smirnov test confirmed that all data were normally distributed. One-way analysis of variance (ANOVA) for repeated measures was used to identify the group effect on each variable, and post hoc pairwise comparison with Bonferroni correction was used in cases in which the group effect was significant. The alpha level for statistical significance was set at p<0.05. All analyses were performed using SPSS (SPSS, Inc., Version 13.0, Chicago, IL).

## Results

**Figure 1:**
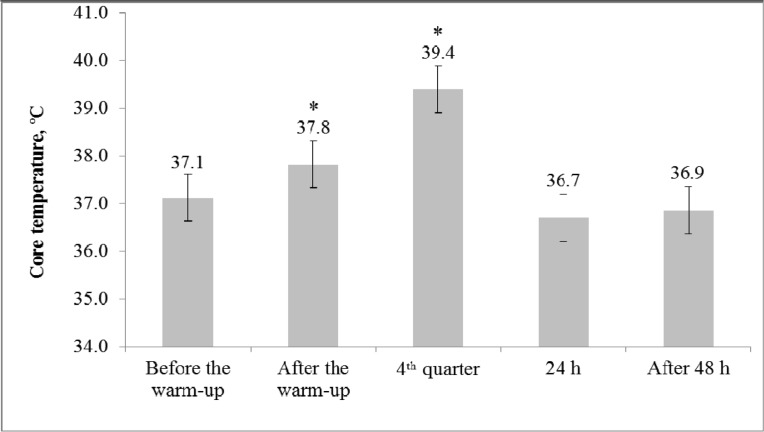
Mean (± SD) values for rectal body temperature ^*^p<0.05 significant difference compared with the values before the warm-up

The rectal body temperature values are presented in Figure 1. Temperature increased significantly after the warm-up (1.9%; F=18.8; η^2^=0.79; p<0.05), continued to increase throughout the game and reached 39.4 ± 0.4ºC after the fourth quarter (F=48.6; η^2^=0.91; p<0.05 compared with the value recorded after the warm-up).

**Figure 2:**
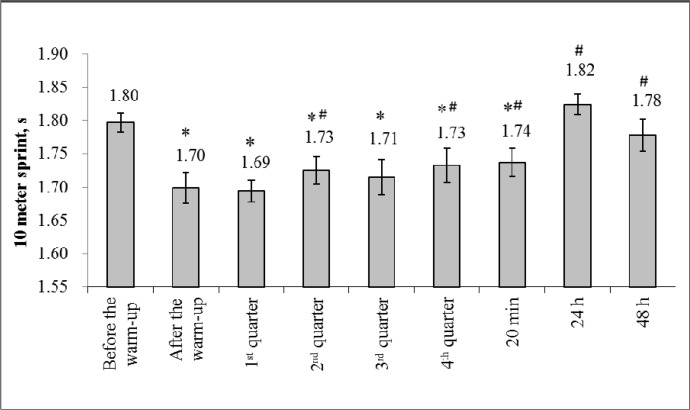
Mean (± SD) values for the 10 m sprint time ^*^p<0.05 significant difference compared with the 10 m sprint time values before the warm-up #p<0.05 compared with the 10 m sprint time values after the warm-up

**Figure 3:**
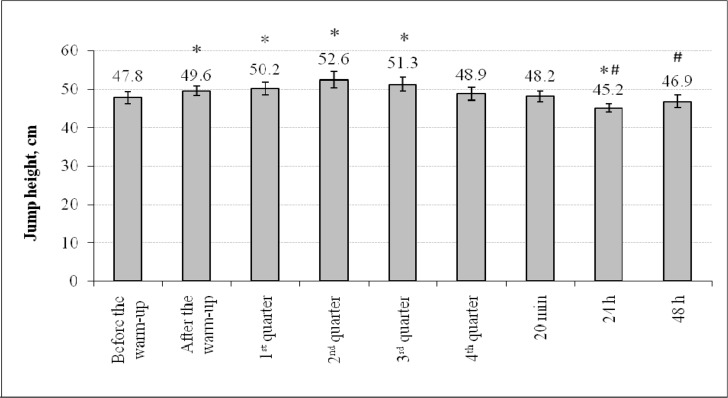
Mean (± SD) values for jump height ^*^p<0.05 significant difference compared with the jump height values before the warm-up #p<0.05 compared with the jump height values after the warm-up

The increase in core body temperature during the warm-up was accompanied by an improvement in the 10 m sprint time (5.5%; F=15.4; η^2^=0.63; p<0.05; Figure 2) and jump height (3.8%; F=6.8; η^2^=0.43; p<0.05; Figure 3). The highest values for the 10 m sprint time and jump height were recorded after the first and second quarter, respectively, whereas for both tests, the worst performance was recorded after the fourth period. Nevertheless, the 10 m sprint time was greater after the fourth quarter when compared with the values recorded before the warm-up (3.3%; F=6.3; η^2^=0.41; p<0.05).

**Figure 4:**
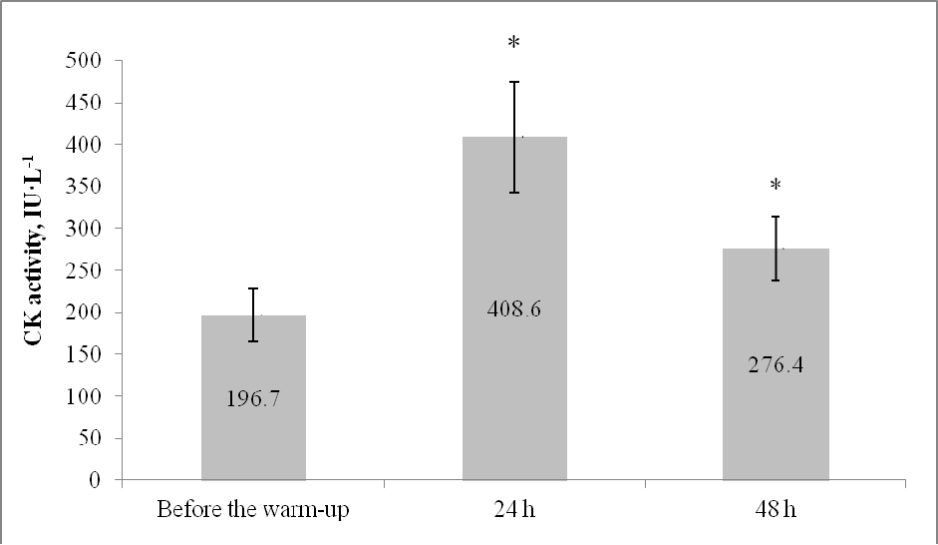
Mean (± SD) values for capillary blood CK activity ^*^p<0.05 significant difference compared with the values before the warm-up

There was a significant increase in CK 24 h (>200%; F=40.2; η^2^=0.89; p<0.05, Figure 4) and 48 h (>30%; F=23.6; η^2^=0.82; p<0.05) after the game, indicating that the physical load induced damage to the players’ muscles. Although there was a trend toward improvement in jump height, these values did not reach the pre-warm-up level within 48 h after the game, a finding that was in contrast with the results for the 10 m sprint time.

## Discussion

In the present study, changes in basketball players’ ability to sprint and jump were investigated using a simulated game. The findings indicate that the players were able to maintain lower limb power up to the fourth quarter, during the major part of the basketball game. Increases in body temperature potentially contributed to enhanced power performance in the initial phases of the game; however, further exertion led to hyperthermia, which may have contributed to the observed decline in performance.

As expected, we found a significant improvement in the basketball players’ jump height and sprint time immediately after the warm-up. The increase in performance matched the increase in body temperature. Furthermore, jump height and sprint time increased in the first half of the game. These findings suggest that for a considerable portion of the game, muscle potentiation mechanisms were predominant over fatigue. Temperature is probably the main performance potentiator, as it may confer a number of psychological effects (Fradkin et al., 2010). The increase in muscle temperature led to an increase in the speed of action potential propagation along the sarcolemma, the activation of ATP hydrolysis, a decrease in muscle viscosity, an increase in elasticity and mobility, and the acceleration of muscle contraction and relaxation (Bishop et al., 2004). Because of the mobilization of the regulatory mechanisms of body function, the energy processes in the body accelerate significantly, which ensures the energy supply to actively functioning structures (Bishop et al., 2004). Interestingly, the players were able to continue to jump very high up to the fourth quarter, though no player substitutions were allowed during the game and therefore manifestation of accumulated effects of fatigue was expected. Similar results were demonstrated previously in competitive basketball players (Castagna et al., 2008; Meckel et al., 2009). These results could be explained by the fact that the game of basketball has an interval character, in which explosions of maximal activity (≤6 s in duration) are followed by rather long (up to 60 s in duration) periods of moderate intensity (Glaister, 2005). If the maximum-intensity work is performed continuously, muscle fatigue occurs much more rapidly compared with the condition in which similar maximum-intensity work is interspersed with rest breaks. For example, while running the 15- to 20-m distance at maximal speed 40 times, the maximal running speed is maintained if 30 s of rest time is allowed after each repetition (Balsom et al., 1992).

After a full game, the sprint and jump performances of the basketball players were impaired compared with the post-warm-up measurements. It is well known that fatigue in response to exercise is related to a number of factors associated with peripheral and central dysfunction (Enoka et al., 2008; Finsterer, 2012). Hence, muscle efficiency loss can be determined by the lack of a sufficient energy supply resulting from inadequate time for ATP and creatine phosphate recovery and from the accumulation of anaerobic glycolysis end products, such as phosphate and hydrogen ions (Ament and Verkerke, 2009; Keyser, 2010). Another possible explanation for fatigue stems from the finding that after the game, the rectal body temperature of the basketball players reached a high value (39.4°C). The literature contains evidence that hyperthermia, which is defined as an elevated body temperature caused by failed thermoregulation, is present when the human body temperature is higher than 38.3°C (Nielsen and Nybo, 2003; Axelrod and Diringer, 2008). With the increase in muscle temperature and the occurrence of hyperthermia, voluntary muscle activation is inhibited as a consequence of a reduced neural drive from the CNS (Martin et al., 2005; Morrison et al., 2004). It is very likely that the hyperthermia observed in the present study contributed to the recorded decrease in the basketball players’ sprint and jump performance.

The effect of fatigue on lower limb power changes was potentially underestimated in the present study because the assessment was completed within 2–4 min after the end of each quarter, which provided some time for recovery. Most of the creatine phosphate and inorganic phosphate recovery occurs within minutes (Yoshida, 1999). Apparently, one should not be surprised that scientists cannot find a great reduction in power indices immediately after the game because even a comparatively small recovery period is quite significant for masking some power deficits. This is rather a systematic limitation in investigations that aim to monitor fatigue using field tests.

The athletes’ jump height was suppressed 24 h after the game. This finding can be related to exercise-induced muscle damage, as twofold increases in CK activity were observed in the blood plasma. The CK activity increase is a widely used indicator of cellular integrity damage (Warren et al., 1999). With regard to exercise-induced damage, sarcolemma, sarcomere and cytoskeletal protein destruction and disrupted Ca2+ regulation are characteristically detected in the muscle fibers consequently resulting in force decline (Lauritzen et al., 2009). Our data are in agreement with previous findings that basketball game induces rather minor muscle damage with minor effects on performance during recovery (Moreira et al., 2014). However, even limited damage may be a sufficient reason to reconsider selection of physical loads on the next day after a basketball game. Maximal intensity loads should be avoided to provide adequate time for muscle power generating machinery to recover completely.

The simulated game approach in the present study could be acknowledged as a limitation, despite the fact that the competitive level of the investigated game was rather high because the players were competing for their role in the team before upcoming college and national championships. It has been shown previously that official games induce greater exertion than the simulated friendly games, possibly due to additional motivation related with more intensive rivalry (Moreira et al., 2012a; Moreira et al., 2012b). Furthermore, examination of a larger number of games may have provided a better vision of the changes, while in the literature a single game analysis is presented quite frequently (Cortis et al., 2011; Moreira et al., 2014). These factors should be kept in mind when interpreting the results of the current study.

## Conclusion

The basketball players’ sprint and jump performance appear to be at least in part associated with body temperature changes, which might contribute to counteract fatigue during the larger part of a basketball game. The impact of exercise-induced muscle damage on lower limb power performance was less severe but evident during the prolonged recovery period (48 h). This finding has to be taken into account when predicting basketball players’ output both during a game and during recovery.
